# A case report of simultaneous PML-IRIS during corticosteroids tapering in a patient with an anti-synthetase syndrome

**DOI:** 10.12688/f1000research.2-283.v1

**Published:** 2013-12-23

**Authors:** Guillaume Martin-Blondel, David Brassat, Hervé Dumas, Emmanuelle Uro-Coste, Daniel Adoue, Hans Lassmann, Michel Clanet

**Affiliations:** 1 Department of Infectious and Tropical Diseases, Toulouse University Hospital, Toulouse, France; 2 INSERM UMR 1043, Centre de Physiopathologie, Toulouse-Purpan, France; 3 Université Toulouse III, Toulouse F-31000, France; 4 Department of Neurology, Toulouse University Hospital, Toulouse, France; 5 Department of Neuroradiology, Toulouse University Hospital, Toulouse, France; 6 Department of Pathology, Toulouse University Hospital, Toulouse, France; 7 Department of Internal Medicine, Toulouse University Hospital, Toulouse, France; 8 Center for Brain Research, Medical University of Vienna, Vienna, Austria

## Abstract

We report a case of simultaneous progressive multifocal leukoencephalopathy-associated immune reconstitution inflammatory syndrome (PML-IRIS) during corticosteroid tapering in a patient with an anti-synthetase syndrome. We describe the challenges associated with the diagnosis and the management of this emerging inflammatory neurological condition in this immunocompromised patient with a severe rheumatic disease. We highlight that, in the setting of IRIS, the low-level of the JC virus viral load requires a sensitive PCR assay before excluding PML.

## Introduction

Progressive multifocal leukoencephalopathy (PML) is a devastating disease due to reactivation of the Polyomavirus JC virus (JCV) in immunocompromised patients ^1^. PML has been associated with immune reconstitution inflammatory syndrome (IRIS) during immune recovery of HIV-infected patients treated by antiretroviral therapy, or in non HIV-infected patients after the withdrawal of therapeutic monoclonal antibodies ^2, 3^. Here we describe a case of simultaneous PML-IRIS during corticosteroids tapering in a patient with an antisynthetase syndrome (ASS).

## Case report

**Figure 1.**
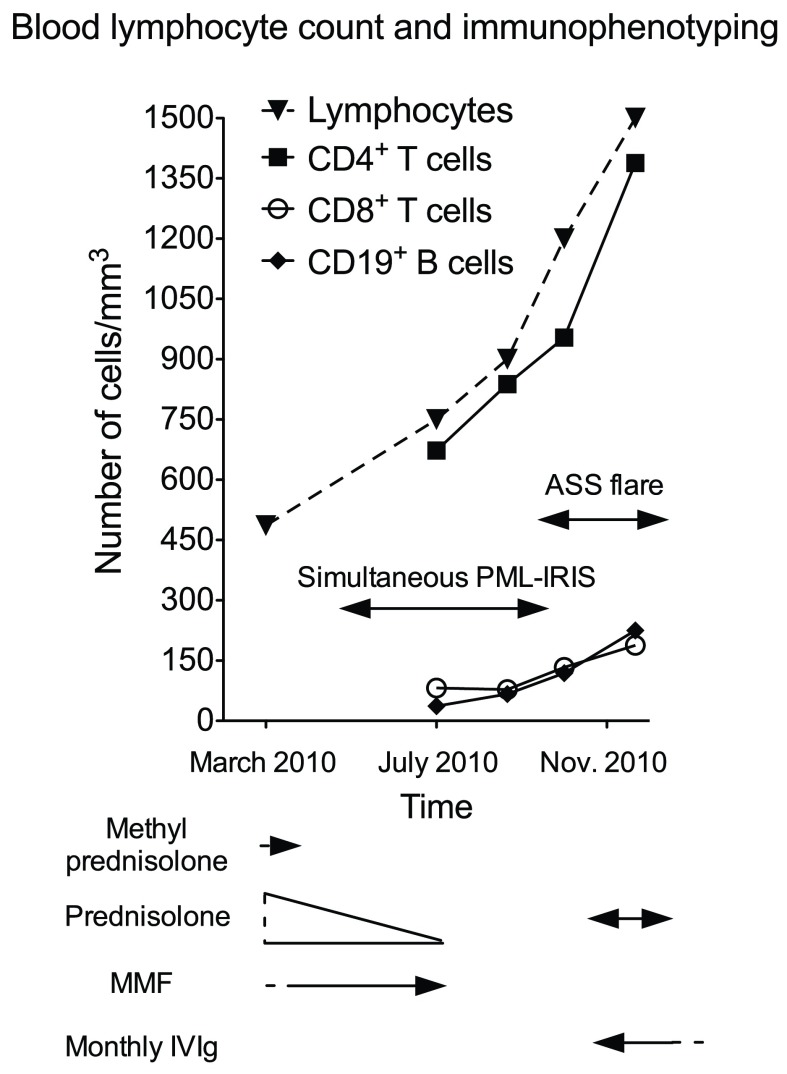
Course of blood lymphocytes according to symptoms and therapy. Arrows underneath the graph represent treatment periods. Single arrow heads represent treatments that were begun or stopped outside of the time period represented on the graph. ASS: Anti-synthetase syndrome; MMF: mycophenolate mofetil.

**Figure 2.**
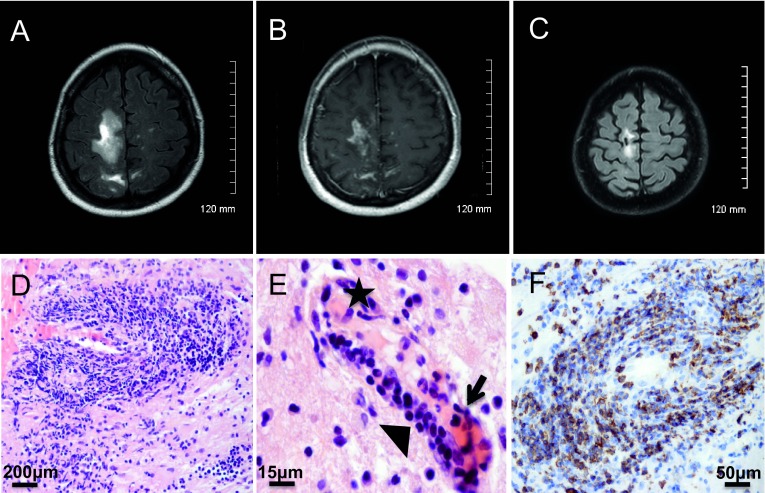
Brain MRI and histopathology. **A**– **B**: Brain MRI at the onset of the simultaneous PML-IRIS showing white matter hyperintensities of the right frontal and parietal region (A, axial T2-WI) with multiple foci of gadolinium enhancement (B, axial T1-WI). **C**: Brain MRI nine months after the onset of PML-IRIS showing sequellar lesions (axial T2-WI). **D**– **E**: Histopathology showing severe perivascular infiltrates (D, H&E × 200) with a vasculitic component (E, H&E × 630, star: fibrin deposit, arrow: neutrophils and serohematic material, arrowhead: lymphocytes infiltrating vessel wall). **F**: Immunohistochemistry analysis (×400) showing perivascular inflammatory infiltrates positive for CD3 antibody (clone F7.2.38, DAKO).

A 62-year-old right-handed French Caucasian woman was diagnosed in October 2007 for an ASS and treated by corticosteroids (methylprednisolone 40 mg/week and prednisolone 20 mg/day) and mycophenolate mofetil (3 g/day). In March 2010 white blood cell count showed profound lymphopenia (486/mm ^3^, normal range 1,500 to 4,000/mm ^3^, Figure 1). Because the ASS was controlled, methylprednisolone was stopped and prednisolone was progressively tapered to 7.5 mg/day. In May 2010 she presented progressive cognitive impairment, followed by a brisk worsening in July 2010 with dizziness and falls. At admission on July 8 ^th^ 2010 neurological examination revealed mental slowness, attention and memory troubles, and paresis of the left lower limb. Brain axial T2-WI MRI revealed confluent subcortical white matter hyperintensities of the right frontal and parietal region ( Figure 2A). Axial T1-WI MRI displayed hypointensities with multiple foci of gadolinium enhancement ( Figure 2B). Prednisolone and mycophenolate mofetil were stopped. The differential diagnoses were viral encephalitis, tuberculosis, cerebral lymphoma, paraneoplastic disorder and CNS involvement of a connective tissue disorder. General examination did not demonstrate any activity of the ASS. Blood cell count showed 750 lymphocytes/mm ^3^ ( Figure 1). Cerebrospinal fluid (CSF) examination on day 2 was normal, and in-house PCR ( *Herpesviridae*, enterovirus, JCV, BK virus, *Toxoplasma gondii* and *Mycobacterium tuberculosis*), and serologies (HIV, *Borrelia* and syphilis), were negative, as well as direct staining and cultures for bacteria and fungi. Blood immunophenotyping showed 673 CD4 ^+^ T cells/mm ^3^ (normal range 500–1,500), 82 CD8 ^+^ T cells/mm ^3^ (normal range 250–950) and 37 CD19 ^+^ B cells/mm ^3^ (normal range 100–600) ( Figure 1). The level of anti-Jo1 antibodies previously detected (Nov 2009, 7.4 AI (normal range 0–0.9) was decreasing (5.9 AI) and a screening for anti-neutrophil cytoplasmic and onconeuronal antibodies was negative. A computed tomography scan showed steady lung interstitial infiltrates, and no evidence for sarcoidosis, tuberculosis or cancer. A stereotactic brain biopsy of the right parietal lobe was performed on July 16 ^th^ 2010. Neuropathological examination showed demyelinated lesions with axonal loss and a severe inflammatory reaction with a vasculitic component and endothelial damage ( Figure 2D–E). Perivascular and parenchymal inflammatory infiltrates showed a pronounced CD3 ^+^ T cell infiltrate ( Figure 2F) composed mostly by CD4 ^+^ T cells, in association with a few CD68 ^+^ macrophages/microglial cells and CD138 ^+^ plasma cells. Anti-Simian virus 40 (SV40) immunohistochemistry (cross-reacting with the JCV) was positive, and a second aliquot of the CSF taken on day 2, sent to Dr. Major’s laboratory at the NIH, was positive for JCV by Real-time *Taq*Man PCR at a low level (23 copies/ml) ^4^, both firmly establishing the diagnosis of PML. A diagnosis of simultaneous PML-IRIS with vasculitis was made. As in the meantime her neurological status had stabilized, the patient did not receive corticosteroids. When followed up in November 2010, mental slowness and paresis of the left lower limb had completely recovered, and repeated MRI showed improvement of previous lesions. In parallel, blood immunophenotyping showed partial normalization ( Figure 1). However, concomitantly she presented with a severe flare of the ASS, with myositis, polyarthritis and active interstitial pneumonitis. A one-week course of oral corticosteroids was initiated together with monthly intravenous polyclonal immunoglobulin therapy (IVIg). By December 2012 the ASS was considered under control with IVIg alone. The patient was fully independent without any neurological abnormalities, while MRI showed sequellar lesions ( Figure 2C).

## Discussion

The diagnosis of PML in this immunocompromised patient is firmly established by the detection of JCV DNA in the CSF and of viral proteins on the brain biopsy sample, and by the exclusion of alternative infections or tumors ^1^.

However, this PML case is associated with very unusual inflammatory features, as attested by contrast enhancement on brain MRI and T cell infiltrates with a vasculitic component on brain biopsy. Because the level of immunosuppression was recently alleviated in this patient, as suggested by the increase of the blood lymphocyte count at admission, we believe that this patient developed a simultaneous PML-IRIS. The development of neurologic abnormalities due to an unusual inflammatory form of PML in the setting of immune recovery is consistent with the definition of simultaneous IRIS ^5^. IRIS results from the restoration of an antimicrobial immune response that causes disproportionate tissue damage in infected organs ^6^. In this case, the corticosteroid tapering, by restoring partially immune surveillance, might have unleashed the T-cell mediated immune response underlying PML-IRIS. The subsequent control of the viral replication might explain the low-level of the CSF viral load in this patient, highlighting that a sensitive PCR assay is required to exclude PML in the setting of IRIS.

Brain infiltrates were mainly composed of CD4 ^+^ T cells, which is another unusual feature of this case. Indeed, a clear dominance of CD8 ^+^ T cells in infiltrates has been observed in natalizumab-associated PML-IRIS in patients with multiple sclerosis (MS) ^7^, and in PML-IRIS in HIV-infected patients ^8^. Nevertheless, a recent case report suggested a central role for CD4 ^+^ T cells in natalizumab-associated PML-IRIS in a patient with MS ^9^. The fact that lymphopenia in our patient mainly relies on CD8 ^+^ T cells, and not on CD4 ^+^ T cells, conversely to the situation in HIV-infected patients, might in part explain this phenomenon.

Despite severe neurological deterioration, this PML correlates with favorable outcome without corticosteroid treatment. The inflammatory reaction associated with IRIS is often self-limited and does not seem to alter survival of patients with PML ^10^. A better control of viral replication might also have contributed to this positive outcome. Because corticosteroids have a profound impact on the JCV-specific T-cell response, they should be reserved for life-threatening PML-IRIS ^11^. Finally when the ASS relapsed, the IVIg therapy was a suitable way to manage the risks of immunosuppression ^12^.

In conclusion, PML-IRIS might occur in patients with rheumatic diseases not receiving therapeutic monoclonal antibodies when immunosuppression is alleviated. PML presentation is unusual in this setting, and diagnosis requires a sensitive PCR assay, and/or brain biopsy.

## Consent

Written informed consent for publication of their clinical details and clinical images was obtained from the patient.
