# Supplementary material for: A case report of simultaneous PML-IRIS during corticosteroids tapering in a patient with an anti-synthetase syndrome
Source: F1000Res. 2013 Dec 23;2:283. [Version 1] doi: 10.12688/f1000research.2-283.v1 (PMC3968897; doi:10.12688/f1000research.2-283.v1)
Supplement: Peer review history

# Peer review: Referee response for version 1

**Published:** 2014-03-07

**Authors:** Xin Dang

**Affiliations:** 1 Department of Neurology, Beth Israel Deaconess Medical Center, Boston, MA, USA

The presence of JCV genomic DNA in CSF is the key evidence for making a diagnosis of PML. However, from time to time in our research, CSF samples from diagnosed PML patients could be JCV negative in conventional PCR; only the most sensitive real time PCR can detect the extremely low JC viral load in these CSF samples. For this reason, I suspect that some PML patients may be misdiagnosed. The remarkable work of Dr. Martin-Blondel's group will encourage other clinicians to look into a possible PML diagnosis in some neural ataxia cases when occurring in immunosuppressed patients.

I have two suggestions that will improve this work:

1. Based on my own experience, JC viral load may increase to a higher level after the PML lesions become visible. If post PML diagnosis CSF samples are available, the data of the JC viral replication kinetic in this patient will help readers gain a better understanding of how JCV replicates in this PML-IRIS patient.
   
2. This patient also shows cognitive dysfunction, which I suspect may be the symptom of a newly defined JCV associated cerebellar disease called JCV Granule Cell Neuronopathy (JCVGCN). Unfortunately, the MRI of the cerebellum is not available in this paper and the authors didn't look into the presence of GCN type JCV strains. However this won't hurt the integrity of this paper. 

I have read this submission. I believe that I have an appropriate level of expertise to confirm that it is of an acceptable scientific standard.

# Peer review: Referee response for version 1

**Published:** 2014-03-03

**Authors:** Joseph Berger

**Affiliations:** 1 Department of Neurology, University of Kentucky, Lexington, KY, USA

The authors describe a fascinating patient with ASS and PML-IRIS.  The latter appeared to develop concomitant with tapering of corticosteroid therapy. I would suggest the following to improve the manuscript:

1. The focus of the paper should be expanded or modified. The occurrence of PML-IRIS at the time of the initial diagnosis of PML is a well-described phenomenon in patients being treated with natalizumab. In fact, as many as 40% of the them have IRIS concomitant with their PML.  This literature should be cited. More importantly, this appears to be the first case of ASS and PML.  There is at least one case report of polymyositis and PML, but I could not find another of ASS and PML and I think that is probably more important than the concomitant appearance of PML and PML-IRIS.
   
2. The figures should include some histopathological sections to demonstrate the demyelination and the SV40 staining. Were bizarre astrocytes and enlarged oligodendroglial nuclei not observed? If so, it should be described.
   
3. Does ASS ever occur with vasculitis or brain disease? A discussion of this aspect of the differential diagnosis would be helpful to the reader.
   
4. The authors reserve comment on the specific clinical features of the rheumatological condition to the very end of their case description. I think it would be more useful to incorporate it earlier in their description.  When did it develop, what were the clinical features, etc?
   
5. Do the authors think that the mycophenolate mofetil contributed to the PML? After all, it carries a black box warning in the U.S. for PML.
   
6. Change the wording from "mainly relies on CD8" to "mainly characterized by CD8" or something similar.
   
7. This patient had resolution of the IRIS in the absence of corticosteroid treatment. This is another important point as the neurological community has widely embraced employment of steroids at the outset of the disorder.  A discussion of this dilemma can be found in Berger, JR (2009).

I have read this submission. I believe that I have an appropriate level of expertise to confirm that it is of an acceptable scientific standard, however I have significant reservations, as outlined above.

# Peer review: Referee response for version 1

**Published:** 2014-02-10

**Authors:** Gabriele Arendt

**Affiliations:** 1 Department of Neurology, University Hospital of Düsseldorf, Düsseldorf, Germany

The authors present an article on a patient with an antisynthetase syndrome developing progressive multifocal leucoencephalopathy = PML-IRIS under cortisone tapering. The case is interesting, well presented and important, because it shows that IRIS-PML might occur also when a relatively mild immunosuppression is reduced.

I have read this submission. I believe that I have an appropriate level of expertise to confirm that it is of an acceptable scientific standard.
